# Effects of Inspiratory Muscle Training in Type 2 Diabetes: A Systematic Review

**DOI:** 10.21470/1678-9741-2022-0366

**Published:** 2023-06-14

**Authors:** Nathalea Spode de Arruda, Náthali de Mello Peixoto, Carine Cristina Callegaro, Maria Elaine Trevisan, Rodrigo Boemo Jaenisch

**Affiliations:** 1 Postgraduate Program in Physical-Motor Rehabilitation, Universidade Federal De Santa Maria, Santa Maria, Brazil.; 2 Human Communication Disorders Postgraduate Program, Universidade Federal de Santa Maria, Santa Maria, Rio Grande do Sul, Brazil; 3 Movement and Rehabilitation Sciences Postgraduate Program, Universidade Federal de Santa Maria, Santa Maria, Rio Grande do Sul, Brazil; 4 Physiotherapy and Rehabilitation Department, Universidade Federal de Santa Maria, Santa Maria, Rio Grande do Sul, Brazil

**Keywords:** Diabetes, Breathing Exercise, Heart Rate Variability, Respiratory Muscle Training

## Abstract

**Introduction:**

People with type 2 diabetes mellitus present multiple complications and comorbidities, such as peripheral autonomic neuropathies and reduced peripheral force and functional capacity. Inspiratory muscle training is a widely used intervention with numerous benefits for various disorders. The present study aimed to conduct a systematic review to identify inspiratory muscle training effects on functional capacity, autonomic function, and glycemic indexes in patients with type 2 diabetes mellitus.

**Methods:**

A search was carried out by two independent reviewers. It was performed in PubMed®, Cochrane Library, Latin American and Caribbean Literature in Health Sciences (or LILACS), Physiotherapy Evidence Database (PEDro), Embase, Scopus, and Web of Science databases. There were no restrictions of language or time. Randomized clinical trials of type 2 diabetes mellitus with inspiratory muscle training intervention were selected. Studies’ methodological quality was assessed using PEDro scale.

**Results:**

We found 5,319 studies, and six were selected for qualitative analysis, which was also conducted by the two reviewers. Methodological quality varied - two studies were classified as high quality, two as moderate quality, and two as low quality.

**Conclusion:**

It was found that after inspiratory muscle training protocols, there was a reduction in the sympathetic modulation and an increase in functional capacity. The results should be carefully interpreted, as there were divergences in the methodologies adopted, populations, and conclusions between the studies evaluated in this review.

**Table:** 

Abbreviations, Acronyms & Symbols				
6WT	= Six-minute walk test		M/F	= Male/female
ANS	= Autonomic nervous system		MIP	= Maximum inspiratory pressure
BMI	= Body mass index		PEDro	= Physiotherapy Evidence Database
CG	= Control group		RCT	= Randomized clinical trial
GLUT-4	= Glucose transporter type 4		SR	= Systematic review
HbA1c	= Glycated hemoglobin		T2DM	= Type 2 diabetes mellitus
IG	= Intervention group		TUG	= Timed Up and Go
IMT	= Inspiratory muscle training		VO₂max	= Maximal oxygen consumption
LFn	= Normalized low frequency			

## INTRODUCTION

Diabetes mellitus is a chronic disease that affects 380 million people worldwide and is related to morbidity and mortality^[1,2]^. Type 2 diabetes mellitus (T2DM) is associated with insulin resistance, which determines persistent hyperglycemia and systemic inflammation^[3,4]^.

Due to hyperglycemia, patients with T2DM have a number of comorbidities and complications, such as an increased risk of developing acute myocardial infarction, coronary artery disease, arterial hypertension, peripheral neuropathies, and changes in the autonomic nervous system (ANS)^[5,6]^. Furthermore, individuals with T2DM may have a reduction in skeletal muscle strength^[7]^, with a consequent reduction in exercise tolerance and ventilatory efficiency^[8]^, which may contribute to a reduction in functional capacity^[9,10]^.

Aerobic and/or resistance exercises provide several benefits in diabetic patients, such as improving insulin sensitivity, reducing cardiovascular risk^[11]^, and improving baroreflex modulation and cardiovascular function^[12]^. Ventilatory exercise modalities, such as inspiratory muscle training (IMT) provide, through increased strength and ventilatory muscle resistance, increased maximal oxygen consumption (VO₂max)^[13]^. In addition, IMT in other conditions, reduces the perception of ventilatory effort, increases resistance to fatigue^[14]^, improves autonomic function by reducing sympathetic modulation and increasing vagal or parasympathetic modulation^[15,16]^, as well as increases functional capacity and quality of life^[17]^.

Studies with IMT and patients with T2DM verified a reduction in glycemic indexes^[18]^, improves autonomic control^[19]^ and functional capacity^[13]^. However, there is not a consensus, through a systematic review (SR), which is the most assertive way to prove impact and effect quality regarding IMT in patients with T2DM. Thus, the present study carried out an SR that verified the IMT effects in patients with T2DM, analyzing glycemic indexes, sympathetic and parasympathetic modulation, and functional capacity.

## METHODS

This SR was conducted following the methodological guidelines of the Preferred Reporting Items for Systematic Reviews and Meta-Analyses (or PRISMA)^[20]^ and following the Cochrane Handbook for Systematic Reviews of Interventions version 6.1 instructions^[21]^. It was registered in the International Prospective Register of Systematic Reviews (or PROSPERO) under the number CRD 42020187090.

Inclusion and exclusion criteria were based on Population, Intervention, Comparison, and Outcome (or PICO) questionnaire model^[22]^, where: Population was T2DM patients, Intervention was IMT and/or respiratory muscle training, Comparator was the comparison of the intervention group (IG) with a control group (CG) without training or with reduced load, and Outcomes were glycemic levels, sympathetic and parasympathetic modulation, and functional capacity. Randomized clinical trials (RCT) were included in this SR. Studies where patients had prediabetes, gestational or childhood diabetes, severe comorbidities, other types of breathing/ventilatory exercises without a description of the IMT protocol, crossover studies, and studies in animal models were excluded. Besides, duplicated studies in databases were excluded as well.

A search was carried out in PubMed®, Physiotherapy Evidence Database (PEDro), Cochrane Library, Embase, Scopus, Web of Science, and Latin American and Caribbean Literature in Health Sciences (or LILACS) databases until March 2020. There were no date and language restrictions. To update the database, another search was carried out in March 2022.

Association of the following intervention descriptors “Inspiratory muscle training” OR “Ventilatory muscle training” OR “Respiratory muscle training” OR “Respiratory exercise” OR “Inspiratory exercise” OR “Threshold IMT” OR “Threshold” OR “Ventilatory exercise” OR “Breathing exercises” OR “Power breath” OR “Inspiratory resistance” OR “Inspiratory muscle loaded” to the outcome descriptor “Diabetes Mellitus” was used in the search strategy.

The articles’ selection was performed by two independent reviewers. If there was a disagreement, a third reviewer was consulted. In the first phase, all studies were evaluated by their titles and abstracts through the EndNote™ X9 software for Windows® 10, the same process was used in the evaluation of the full text.

After a complete reading of the included studies, a protocol for data extraction was established and performed using the Excel® for Windows® 10. The two reviewers extracted and organized the following data referring to IG and CG: authors, title, year, journal, sample characteristics, description of the intervention (intensity, application time, repetitions, series, protocol duration, weekly frequency, equipment used, and load), evaluated outcomes, and results description.

Aiming to identify the articles’ methodological limitations, the PEDro quality scale was used, by the two reviewers, for RCTs, where the maximum score is 10 points. It were considered high quality (> 7 points), moderate quality (6 or 5 points), and low quality (≤ 4 points)^[23]^.

## RESULTS

Studies Selection

**Fig. 1:**
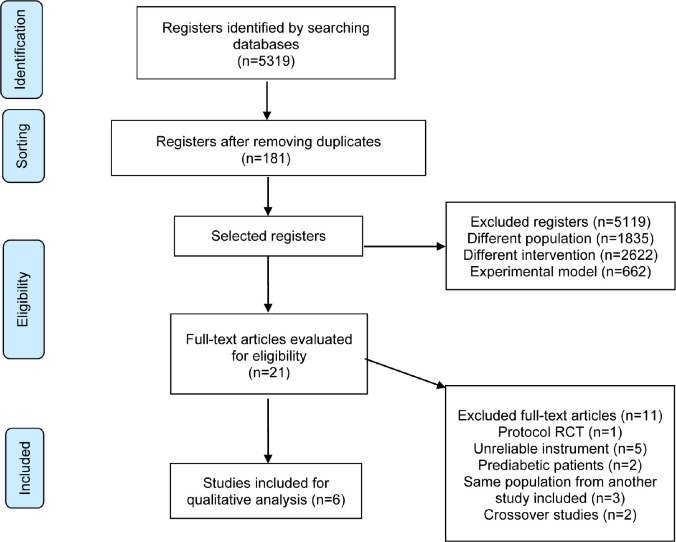
Preferred Reporting Items for Systematic Reviews and Meta-Analyses (or PRISMA) flowchart of included articles. RCT=randomized clinical trial.

It was found 5,319 references in the databases, 181 of them were excluded because they were duplicates. From the analysis of titles and abstracts, 5,119 studies were excluded and 21 were selected for full text reading. After reading them completely, six studies were eligible for this SR (Figure 1).

Methodological Quality

The studies were evaluated using PEDro scale, showing variation in the levels of methodological quality. Two studies were classified as high quality, two studies as moderate quality, and two as low quality (Table 1).

**Table 1:** Methodological quality.

Assessed articles	1	2	3	4	5	6	7	8	9	10	11	Total
Ahmad, 2020	X			X				X		X	X	4
Albarrati, 2020	X	X	X	X				X	X	X	X	7
Correa, 2011	X	X		X	X		X			X	X	6
Kaminski, 2015	X	X		X						X	X	4
Mowad, 2020	X	X		X				x		X	X	5
Pinto, 2021	X	X	X	X	X		X	X	X	X	X	9

Criteria: 1, eligibility; 2, random allocation; 3, hidden allocation; 4, comparison with basic characteristics; 5, blinding of subjects; 6, blinding of therapists; 7, blinding of the evaluators; 8, description of patient follow-up; 9, analysis of intention to treat; 10, comparison between groups; 11, point estimates and variability

Participants’ Characteristics

One hundred and seventy participants were included in the sample (IG: 83, CG: 87), which was composed of adults, with an average age between 42 and 63 years. Most were pre-obese, except for one study that included obese patients (Table 2).

**Table 2:** Sample characteristics.

Study	N	Gender M/F	BMI	Presence of neuropathy	MIP (cmH₂O)	Diabetes duration (years)	Dropouts
Ahmad, 2020	IG: 12	0/28	IG: 34,6±4,6	Not mentioned	Not mentioned	IG: 4±3	IG: 2
	CG: 14		CG: 36,8±5,7			CG: 36,8±5,7	
Albarrati, 2020	IG: 15	66%/33%	IG: 29,85±4,53	Not mentioned	IG: 76,33±9,5	Not mentioned	Not mentioned
	CG:15		CG: 29,46±4,46		CG: 70,1±7,7		
Correa, 2011	IG: 12	IG: 7/5	IG: 27,3±3,2	Yes	IG: 56±13	IG: 11,6 ±4,7	IG: 5
	CG: 13	CG: 5/8	CG: 28,2±2,6		CG: 52±10	CG: 13,9±8,3	CG: 3
Kaminski, 2015	IG: 5	Not mentioned		Yes	IG: 98±34	IG: 13 ± 1	N=2
	CG: 5				CG: 88±26	CG: 10,7 ± 6	
Mowad, 2020	IG: 28	IG: 20/8	GI: 29,2±3,7	Yes	IG: 56±13	Not mentioned	IG: 2
	CG: 27	CG: 22/5	CG: 27,9±4,8		CG: 52±10		CG: 3
Pinto, 2021	IG: 11	8 (26,7)	IG: 28,5±3,2	5 (16,7)	IG: 90,4±4,2	9 (5-12,7)	IG: 4
	CG: 13		CG: 27±3,1		CG: 98,8±5,3		CG: 2

Data are expressed as n, %, mean ± standard deviation, and median (P25.75) BMI=body mass index; CG=control group; IG=intervention group; M/F=male/female; MIP=maximum inspiratory pressure

Intervention Description and Results

In the protocols, IMT loads ranged between 30% and 75% of the maximum inspiratory pressure (MIP) for at least eight weeks, with a frequency between three and seven times a week (Table 3). Considering six RCTs, three showed an increase in MIP and did not assess muscle endurance^[13,19,24]^. One showed an increase in MIP and endurance^[25]^ and one obtained an increase only in muscular endurance, with no difference in MIP after the protocol^[26]^.

**Table 3:** Interventions description.

Study	Outline	Duration (weeks)	Device type	Frequency, duration (time per week in minutes)	Load (% MIP)	CG activity	Intervention details
Ahmad, 2020	RCT	8	Threshold IMT	5×/week, 15-25 min (supervised)	30%	No exercise	Deep, slow breaths in a diaphragmatic pattern
Albarrati, 2020	RCT	8	Threshold IMT	7×/week, 30 min (1×/week supervised)	40%	15%	Weekly assessment, diaphragmatic breathing, 15 to 20 breaths/min
Correa, 2011	RCT	8	Threshold IMT	7×/week, 30 min (6×/week at home and 1× supervised)	30%	Minimum load (7 cmH₂O)	Weekly assessment, diaphragmatic breathing, 15 to 20 breaths/min
Kaminski, 2015	RCT	8	Threshold IMT	7×/week, 30 min	30%	No load	Weekly assessment, diaphragmatic breathing, 15 to 20 breaths/min
Mowad, 2020	RCT	12	TRAINAIR®	3×/week, 30 min (supervised)	75%	10%	6 cycles of 30 breaths
Pinto, 2021	RCT	12	POWER®breathe	7×/week, 30 min (1×/week supervised)	30%	2%	Weekly assessment, diaphragmatic breathing, 15 to 20 breaths/min

CG=control group; IMT=inspiratory muscle training; MIP=maximum inspiratory pressure; RCT=randomized clinical trial

One RCT showed low methodological quality (PEDro score 4), used a MIP load of 30%, and observed a reduction in sympathetic modulation after protocol^[19]^. In one study, there was a reduction in glycemic levels in eight weeks of protocol^[26]^. Two studies demonstrated that IMT increased functional capacity, which was assessed by the six-minute walk test (6WT)^[24]^, and VO2max, assessed by the cardiopulmonary exercise test^[13]^. They were considered high-quality (> 7 points) and moderate-quality (6 or 5 points) studies, respectively (Table 4).

**Table 4:** IMT in the glycemic index, autonomic function, and functional capacity.

Study	PEDro score	Diaphragmatic muscle strength	Glycemic index	Autonomic function	Functional capacity
Ahmad, 2020	4	Not mentioned	There was no statistical difference	Not assessed	Not assessed
Albarrati, 2020	7	Increased MIP	Not assessed	Not assessed	Increase in 6WT covered distance, TUG time, and palm grip strength
Correa, 2011	6	Increased MIP	Not assessed	There was no statistical difference	There were no significant results
Kaminski, 2015	4	Increased MIP	Not assessed	Reduction of the sympathetic component (LFn)	There were no significant results
Mowad, 2020	5	Increased MIP	Not assessed	Not assessed	Increased VO₂max
Pinto, 2021	9	Increased endurance	Reduced glycemic levels after 8 weeks of IMT at 30% MIP, with no difference after 12 weeks, neither in HbA1c	Not assessed	Not assessed

6WT=six-minute walk test; HbA1c=glycated hemoglobin; IMT=inspiratory muscle training; LFn=normalized low frequency; MIP=maximum inspiratory pressure; PEDro=Physiotherapy Evidence Database; TUG=Timed Up and Go; VO₂max=maximal oxygen consumption

## DISCUSSION

To the best of our knowledge, the current SR was the first to evaluate IMT effects in patients with T2DM. Six studies were qualitatively analyzed. Regarding the evaluated outcomes, one study demonstrated to reduce the glycemic levels of patients with T2DM^[26]^ and one study verified the reduction of the normalized low frequency (LFn) (sympathetic component)^[19]^. Four studies analyzed the IMT effect on functional capacity of patients with T2DM, where two RCTs showed an increase in functional capacity^[13,24]^.

The diabetic population has skeletal muscle impairment caused by endothelial wall injuries and reduced muscle capillary density^[27]^. Due to this, patients with T2DM may have reduced inspiratory muscle strength^[7]^. When we analyzed the ventilatory strength and endurance, we found that among the six RCTs, three showed an increase in MIP after IMT, without endurance assessment^[13,19,24]^. One RCT did not assess MIP^[28]^, and another one found an increase in muscle endurance with no difference in MIP^[26]^. This happened probably because patients did not have ventilatory weakness and the load was considered mild for this, which may have negatively interfered with this outcome.

IMT leads to beneficial effects on the ANS, specifically on sympathetic and parasympathetic modulation in different populations^[15,16,29,30]^. In patients with T2DM with autonomic dysfunctions, an RCT present in this SR (PEDro score 4) resulted in a reduction in the LFn component (sympathetic modulation) after an eight-week IMT protocol at 30% of MIP, 30 min/day^[19]^. Corrêa et al., however, showed no difference in autonomic modulation in individuals with ventilatory weakness with a similar training protocol (6 as methodological quality)^[25]^.

Patients with T2DM and autonomic dysfunction have impaired nerve blood supply, in other words, nerve conduction affected with consequent neuropathy, being more impaired than those without nerve injury^[31,32]^. Regarding autonomic modulation, one study by our group showed that IMT improved autonomic function in diabetic patients^[33]^ with increased parasympathetic activity. In healthy individuals, the use of IMT with a load of 30% MIP provides an increase in vagal modulation, while higher loads can determine a predominance of sympathetic modulation^[34,35]^.

IMT protocol acute showed a reduction in the normalized high frequency (or HFn) component in one session, with a MIP training session at 60%^[36]^, *i.e.*, there was a greater predominance of the sympathetic component during one training session with that load. Considering this, the IMT intensity provides different effects on the ANS, and its prescription must be carefully performed. In addition, other types of exercise, such as aerobic and resistance exercises, when acutely evaluated, show a reduction in parasympathetic modulation and an increase in sympathetic modulation, through muscle mechanoreceptors, as well as an increase in functional capacity. This mechanism becomes even more noticeable as exercise intensity increases^[37,38]^. On the other hand, long-term physical exercises can provide an increase in vagal modulation in individuals with T2DM^[39]^.

Physical exercises, in the most different modalities, are a non-pharmacological alternative to improve the glycemic response and glycated hemoglobin (HbA1c) levels in diabetic patients^[40-42]^. Furthermore, ventilatory exercises such as controlled and relaxed breathing have already been effective to improve glycemic control^[43]^. This is due to the increase in vagal modulation after controlled exercises, since a stimulus to the hepatic vagus nerve has reduced blood glucose in rats^[44]^. In an acute way, IMT has already shown interference on glycemic levels, demonstrating a reduction in values^[18,36]^.

Furthermore, it has been previously reported that daily training with a MIP load at 40% improves insulin sensitivity and beta cell secretion in elderly non-diabetic patients^[45,46]^. In this way, there is also an improvement in glucose transport after IMT, through greater uptake in glucose transporter type 4 (GLUT-4)^[45]^, similarly to what occurs in aerobic exercises^[40]^. In contrast, an RCT from our SR (PEDro score 9) resulted in a blood glucose reduction in eight weeks of IMT, but not in 12 weeks, or in HbA1c^[26]^, while another RCT (PEDro score 4) found no difference on the glycemic index after eight weeks of training^[28]^. The diaphragm muscle, the main muscle trained with IMT, has smaller motor units than limb muscles in healthy individuals^[47]^, what may be a possible explanation for the non-reduction of glycemic levels after IMT exercise protocols, different from other types of exercise, where muscle recruitment appears to be greater, as well as glucose uptake through GLUT-4.

The practice of physical exercises in the most diverse ways is impaired in individuals affected by T2DM, as they are at greater risk of manifesting muscle fatigue resulting from the disease’s comorbidities, as well as reduced functional capacity^[8,48]^. In this SR, four studies evaluated the IMT effect on physical-functional capacity and performance. Moawd et al., in a study with methodological quality 5, demonstrated an increase in VO2max assessed by the cardiopulmonary test^[13]^. Albarrati et al. verified, through the 6WT, an increase in the distance covered and a reduction on the Timed Up and Go (or TUG) test time (PEDro score 7)^[24]^. Other two studies (PEDro scores 4^[25]^ and 6^[19]^) did not find an improvement for this outcome.

The improvement in functional capacity after IMT can be explained by the diaphragmatic metaboreflex mechanism. When there is fatigue of the diaphragmatic muscles and a consequent accumulation of metabolites, there is a greater coupling of metabolism products to the metaboreceptors, which, in turn, send information to the ANS with consequent sympathetic hyperexcitation. In this way, an increase in sympathetic modulation that determines peripheral vasoconstriction and, consequently, a reduction in peripheral blood flow at the expense of a redirection of blood to the diaphragm. After IMT, there is an increase in ventilatory strength, consequently, there is an increase in the threshold of fatigue perception, that is, of the activation of the metaboreflex, attenuating the effort perception and causing the preservation of blood flow in the periphery during exercise with a consequent increase in functional capacity^[49-51]^.

Therefore, from results in this SR, we can infer that IMT still does not have the necessary scientific support to be considered as an alternative treatment of T2DM patients. Despite this, it can be considered an ally with other exercise categories, considering that in diabetic patients, combined modalities are more effective than just one form of exercise^[52]^, especially when patients are elderly and are not able to perform certain exercises^[45]^.

### Limitations

Regarding this SR limitations, we point out the small number of studies, as well as the reduced samples, in addition to the IMT protocols heterogeneity, designs, and outcomes, facts that did not allow us to carry out a meta-analysis.

## CONCLUSION

This SR analyzed six studies, which evaluated the IMT effect in patients with T2DM. IMT is a non-pharmacological form of treatment that benefits the most diverse populations. Through increasing the strength of diaphragmatic muscles, the performance of other exercises is favored, since the fatigue threshold seems to be increased with consequent improvement in functional capacity. It is also possible to infer a probable improvement in autonomic modulation, depending on the load chosen for the IMT. In patients with T2DM, through our study, we found that IMT can be a tool to improve autonomous modulation and functional capacity and should be combined with other types of exercise; however, the results need to be interpreted with caution because they are still inconclusive. Thus, more RCTs should be carried out to obtain a clearer answer.

**Table:** 

Authors’ Roles & Responsibilities	
NSA	Substantial contributions to the acquisition, analysis, and interpretation of data for the work; revising the work critically; final approval of the version to be published
NMP	Substantial contributions to the acquisition, analysis, and interpretation of data for the work; revising the work critically; final approval of the version to be published
CCC	Revising the work critically; final approval of the version to be published
MET	Revising the work critically; final approval of the version to be published
RBJ	Substantial contributions to the acquisition, analysis, and interpretation of data for the work; revising the critically; final approval of the version to be published
